# Multiobjective design optimization of parabolic trough collectors

**DOI:** 10.1038/s41598-022-24305-3

**Published:** 2022-11-19

**Authors:** Mohamed Mahran Kasem

**Affiliations:** 1 Aerospace Engineering Department, Cairo University, Giza, 12613 Egypt; 2 School of Engineering and Applied Science, Nile University, Shaikh Zayed City, 12588 Egypt

**Keywords:** Engineering, Mechanical engineering

## Abstract

Despite the large amount of research conducted on PTC performance analysis, few and rare numbers of research have considered the design optimization of PTCs. In the present work, a novel multiobjective-optimization model is developed for design optimization of PTCs. The objective functions are the thermal and exergetic efficiencies because they are the most important performance indicators (PIs) of PTCs. The design variables are the inlet temperature, and the outlet and inlet diameters of the PTC receiver tube. The PTC material volume (refers to the volume of the PTC receiver and collector) is kept constant throughout the optimization process to enhance the PTC performance without incurring additional cost (material). A parametric analysis is conducted before the optimization. The inlet-mass flow-rate effect is found to be negligible in contrast to the inlet temperature. Therefore, the latter is considered as a design parameter in the optimization process. Nine thermal fluids are used in the present optimization, which include pressurized water, Therminol, molten salt, liquid sodium, Syltherm, air, carbon dioxide, helium and hydrogen. The present optimization model is found to be efficient in maximizing both the thermal and exergetic efficiencies. Water achieves maximum optimal thermal efficiency, whereas helium achieves maximum optimal exergetic efficiency. Liquid sodium exhibits the best PI (60.725).

## Introduction

According to the statistical review of world energy^1^, “the COVID-19 pandemic had a dramatic impact on energy markets, with both primary energy and carbon emissions falling at their fastest rates since the Second World War. Nevertheless, renewable energy continued to grow, with solar power recording its largest ever increase.” The review also reveals that energy consumption using conventional sources (such as oil) decreased by 4.5% in 2020 in contrast to that using solar and wind energy. With the ever–increasing demand for energy, solar–energy sources have continued to grow and are being widely used. Parabolic trough collectors (PTCs) are among the popular technologies that are used to extract energy from the sun^2^. They are considered as among the best methods for solar–energy harvesting. PTC utilizes the sun energy and apply it to thermal fluids for several usages such as power generation, water desalination^3^, and water heaters^4^. In water desalination, PTC systems are used to transform sea water into fresh water by reducing its salinity concentration^3^. PTCs can also be integrated in cooling systems for home–building cooling or heating^5^. Intensive works that investigate the performance and development of wide range of PTCs can be found in literature.

One of the important component in PTCs is the working fluid because it determines the PTC efficiency as well as the maximum energy that can be extracted from the system. Bellos et al.^6^ investigated the effect of gases as a working fluid on energetic and exergetic PTC performance. They investigated six working fluids which included air, nitrogen, carbon dioxide, helium, neon, and argon. According to them, helium and carbon dioxide are the best working fluids for low- and high-temperature applications, respectively. In 2018, Bellos et al.^7^ investigated seven working fluids for the same purpose. Pressurized water and carbon dioxide were found to be the best working fluids for low- and high-temperature applications, respectively. Zaharil and Hasanuzzaman^8^ studied the energetic and exergetic performance of PTCs. They used six working fluids, namely, pressurized water, Syltherm 800, Therminol VP-1, solar salt, Hitec XL, and liquid sodium. They considered the climate-change effect in their study. They concluded that the increase in ambient temperature improved the energetic efficiency. Liquid sodium was found to demonstrate better performance in the study period than the other working fluids. The use of supercritical fluids was better for thermal-energy storage^8^.

Different mathematical models have been developed for analysis, modeling, and performance study of PTCs. Each mathematical model focused on certain application and performance analysis. Tzivanidis et al.^9^ developed a simple mathematical model for the most well-known PTCs. They employed a mathematical model to validate their simulation and numerical analysis of small PTC under different operating conditions. In addition, they analyzed the heat-transfer phenomena and predicted the PTC efficiency. In the same manner, Bellos and Tzivanidis^10^ developed a nonlinear analytical expression that included the parameters that could affect the PTC performance. The new model was found to have good accuracy. A reduced-order mathematical model^11^ was developed to determine the steady-state heat-transfer performance of PTCs. The reduced model was solved using an appropriate iterative technique to determine the PTC axial and radial temperature profiles.

PTCs can assume different shapes such as V- and dish-shaped PTCs. Customized mathematical models are needed to investigate the performance of these PTC types. Beltran et al.^12^ developed a mathematical model to investigate the thermal performance and optical behavior of a solar parabolic dish. They employed the mathematical model for performance analysis and parametric study of dish collectors including their environmental effects. Bie et al.^13^ developed a novel mathematical model for V-shaped absorber PTC based on a thermal-resistance network method. Their model was validated using experiments under different weather conditions. All these models provided a mathematical basis for performance analysis, parametric study, and design optimization of PTCs.

Different parameters are found to affect the performance and energy capacity of PTCs. Thappa et al.^14^ investigated the effect of a receiver PTC size on its performance. They compared two receiver tubes and determined their effects on the PTC output parameters. They reported a remarkable decrease in the heat loss coefficient with the decrease in the PTC receiver diameter. El-Bakry et al.^15^ examined the effect of adding a radiation heat shield on the PTC efficiency. They found an enhancement in both the thermal and exergetic efficiencies of PTC. They reported an improvement of up to 15.4% and 14.4% in both the energetic and exergetic efficiencies, respectively. Amiri et al.^16^ studied the effect of adding a solar still to PTC. They studied different solar-still parameters such as saline water and absorber for water desalination. Conventional PTCs are exposed to high thermal stresses and deflection due to the concentration of solar irradiation at the absorber-tube bottom. One solution for this problem is to use a rotating absorber tube^17^. Proper selection of the absorber rotating speed can improve the PTC efficiency by 17% and reduce absorber temperature by 60%. The use of steel receiver, glass cover^18^, and hot mirror coating^19^ can improve the PTC efficiency. The presence of glass cover increased the PTC efficiency from 39 to 51%^18^. While the addition of hot mirror coating increased the efficiency up to 7%^19^. Bellos et al.^20^ investigated the use of nanofluids to enhance the PTC efficiency. The use of an oil-based nanofluid enhanced the thermal efficiency by 0.76%, whereas the salt-based nanofluid improved the thermal efficiency by 0.26%.

Nascimento et al.^21^ developed a new algorithm for sizing PTCs in terms of operating temperature and thermal loads. They verified their model using four thermal fluids. Hoseinzadeh et al.^22^ used the Monte Carlo method for geometric optimization of PTCs. The design variables were the rim angle, aperture width, and receiver diameter. Their model could significantly improve the PTC optical efficiency. Kumer and Shukla^23^ developed a model for a PTC design for optimum power. Their model was found efficient in maximizing PTC collection efficiency by selecting optimum values for concentration ratio, focal length, and rim angle Ehyaei et al.^24^ developed a multiobjective optimization model for the design of PTCs. They utilized the swarm optimization method to minimize the production cost and maximize the exergy efficiency. PTC length, width, and internal absorber diameter were defined as design variables. Optimum values of 29.22% and 0.0142 $/kWh were obtained for the exergy efficiency and PTC cost, respectively.

Optimization is a process of maximizing or minimizing certain functions under some preferences. The function to be improved is called objective or cost function, and the preferences are called constraints. Optimization is applied to several engineering, fluid, and structural-design problems^25,26^ for designing stiff and cost-efficient products. Different models and algorithms are used for this purpose^27–29^. The general form of an optimization problem can be expressed as follows:

1 $$ \begin{aligned} & {\text{Find}}\,{\text{design}}\,{\text{variable}}\,{\text{vector}}\,{\vec{\mathbf{x}}} = \left( {{\varvec{x}}_{1} ,{\varvec{x}}_{2} , \ldots ,{\varvec{x}}_{{\varvec{N}}} } \right)^{{\varvec{T}}} ,{\text{which}} \\ & {\text{Minimizes }}\quad {\mathbf{f}}\left( {{\vec{\mathbf{x}}}} \right) \\ & {\text{Subject }}\,{\text{to }}\quad {\varvec{g}}_{{\varvec{j}}} \left( {\vec{\user2{x}}} \right) \le 0,\quad {\varvec{j}} = 1,2, \ldots ,{\varvec{J}} \\ & {\varvec{h}}_{{\varvec{k}}} \left( {\vec{\user2{x}}} \right) = 0, \quad {\varvec{k}} = 1,2, \ldots ,{\varvec{K}} \\ & {\varvec{x}}_{{\varvec{i}}}^{{\varvec{L}}} \le {\varvec{x}}_{{\varvec{i}}} \le {\varvec{x}}_{{\varvec{i}}}^{{\varvec{U}}} ,\quad {\varvec{i}} = 1,2, \ldots ,\user2{N,} \\ \end{aligned} $$

where $\mathbf{f}(\overrightarrow{\mathbf{x}})$ denotes the objective function, ${g}_{j}\left(\overrightarrow{\mathbf{x}}\right)$ is the inequality constraints, and ${h}_{k}\left(\overrightarrow{\mathbf{x}}\right)$ is the equality constraints. ${{{\varvec{x}}}_{{\varvec{i}}}}^{{\varvec{L}}}$ and ${{{\varvec{x}}}_{{\varvec{i}}}}^{{\varvec{U}}}$ represent the lower and upper boundaries (side constraints) of the decision variables, respectively. Equation (1) expresses the general form of a nonlinearly constrained optimization problem^30^.

When two or more objective functions are employed, the problem is called multiobjective-optimization (MOO) problem in which objective function $\mathbf{f}(\overrightarrow{\mathbf{x}})$ comprises two or more cost functions in the form $\mathbf{f}\left(\overrightarrow{\mathbf{x}}\right)=\left({f}_{1}\left(\overrightarrow{x}\right),{f}_{2}\left(\overrightarrow{x}\right),{f}_{3}\left(\overrightarrow{x}\right)\dots {f}_{M}\left(\overrightarrow{x}\right)\right)$, where *M* denotes the total number of objective functions that define a multiobjective space^31^. MOO is a challenging process because enhancement of one function usually degrades the other functions.

Several techniques have been proposed to overcome the MOO challenges. The most popular MOO methods are the Pareto and scalarization or weighting-sum methods^32^. Pareto optimization is based on the concept of obtaining all minimum points that satisfy the design constraints. The resulting optimal solutions are called Pareto-optimal solutions or simply the Pareto front. Pareto front represents a set of optimum solutions for MOO. Let us consider MOO with two objective functions where many solutions represent the optimal value of general objective function $\mathbf{f}\left(\overrightarrow{\mathbf{x}}\right)$. Some solutions improve the performance of one objective function compared with the others. The locus of all optimal points represents the Pareto front.

**Figure 1:**
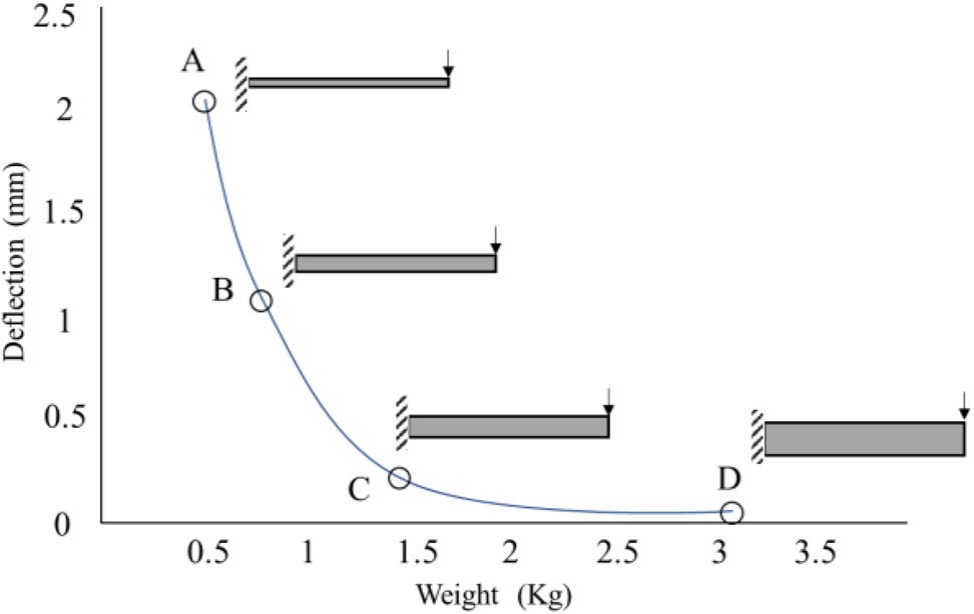
Pareto front for MOO of a beam with minimum deflection and minimum weight^31^.

Figure 1 shows an example of a Pareto-optimal solution for a beam where minimum-deflection and minimum-mass MOOs are employed. Point (A) represents the minimum weight and maximum deflection point, and Point (D) represents the minimum deflection and maximum weight^31^.

Despite the vast amount of works available in the literature that studied and analyzed the PTC performance, few and rare studies on PTC design optimization have been conducted. In the present work, a MOO model is developed for the design and performance enhancement of PTCs. The objective functions are the thermal and exergetic efficiencies. The design variables are the fluid inlet temperature as well as the inlet and outlet diameters of the receiver tube. The total PTC volume is kept constant during the optimization process to improve the PTC performance without any additional cost. Nine thermal working fluids are considered in the present study: five liquids and four gases. A mathematical model is developed using MATLAB, and design optimization is performed to the PTC using the built-in MATLAB genetic algorithm (GA) for MOO. The novelty of the present work includes the development of new MOO model, and the parametric analysis using surface plots.

## Mathematical model

**Figure 2:**
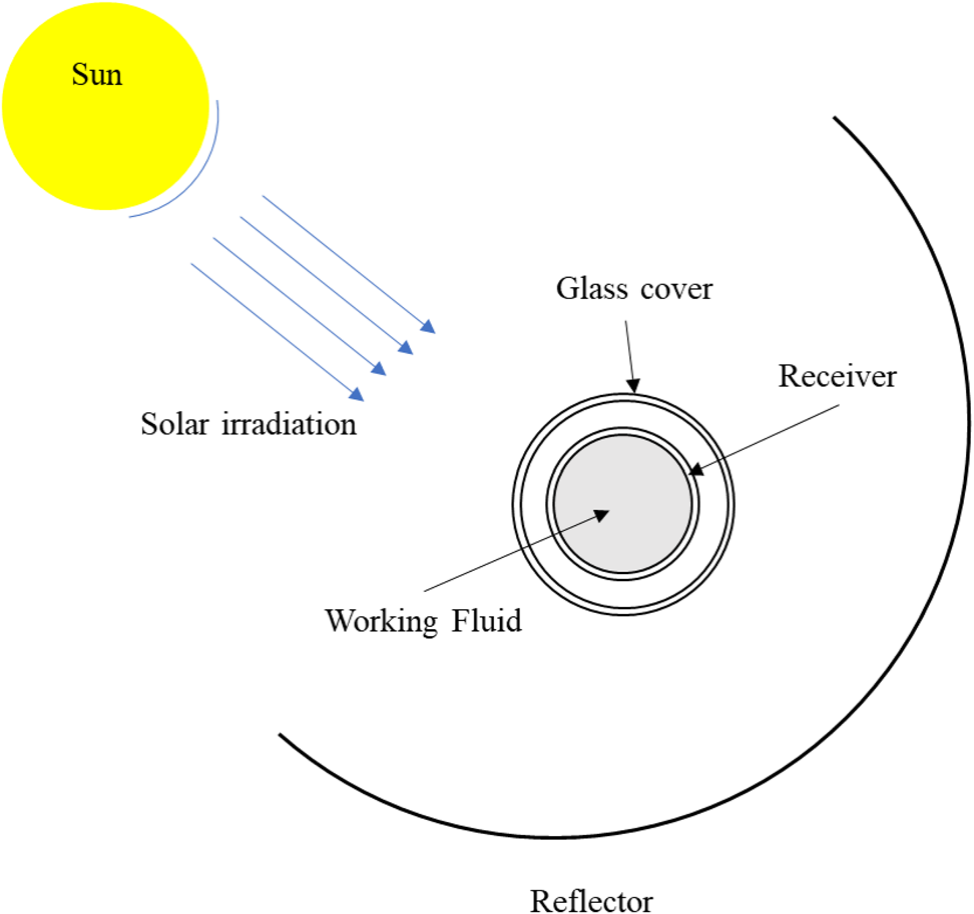
PTC schematic diagram.

A typical PTC is constructed from a receiver tube that contains a heat-transfer fluid, a glass cover, and a reflector. The reflector reflects the solar irradiation to a focal point where the receiver tube is fixed, as shown in Fig. 2. The key idea behind PTC is to harvest the solar energy, increase the working-fluid temperature, and subsequently utilize it to generate power. Unfortunately, PTCs cannot capture all available solar energy. Thus, mathematical models are developed to calculate the amount of thermal energy that can be captured from the sun as well as the PTC efficiency. The available solar energy can be determined as^7^,

2 $${{\varvec{Q}}}_{{\varvec{s}}}={{\varvec{A}}}_{{\varvec{a}}}\boldsymbol{ }\boldsymbol{ }{{\varvec{G}}}_{{\varvec{b}}},$$

where ${G}_{b}$ represents the solar-beam radiation $\left[\frac{\mathrm{W}}{{\mathrm{m}}^{2}}\right]$ and ${A}_{a}$ denotes the aperture area. The amount of useful energy that can be captured by the thermal fluid can be defined as^7^

3 $${{\varvec{Q}}}_{{\varvec{u}}}={{\varvec{m}}}^{{\varvec{o}}}\boldsymbol{ }\boldsymbol{ }{{\varvec{C}}}_{{\varvec{p}}}\boldsymbol{ }\left({{\varvec{T}}}_{{\varvec{o}}{\varvec{u}}{\varvec{t}}}-{{\varvec{T}}}_{{\varvec{i}}{\varvec{n}}}\right).$$

${{\varvec{m}}}^{{\varvec{o}}}$ denotes the mass flow rate, ${{\varvec{C}}}_{{\varvec{p}}}$ defines the specific-heat coefficient at constant pressure, ${T}_{out}$ represents the outlet temperature, and ${T}_{in}$ is the inlet temperature.

The energy-balance equation is a key equation that relates the total solar energy, useful energy, and loss energy; it is expressed as^7^

4 $${{\varvec{Q}}}_{{\varvec{u}}}={{\varvec{Q}}}_{{\varvec{s}}}\boldsymbol{ }{{\varvec{\eta}}}_{{\varvec{o}}{\varvec{p}}{\varvec{t}}}-{{\varvec{Q}}}_{{\varvec{l}}{\varvec{o}}{\varvec{s}}{\varvec{s}}},$$

where ${\eta }_{opt}$ is the optical efficiency, and ${Q}_{loss}$ is the heat loss due to convection and radiation, which can be expressed as^7^,

5 $${{\varvec{Q}}}_{{\varvec{l}}{\varvec{o}}{\varvec{s}}{\varvec{s}}}={{\varvec{A}}}_{{\varvec{c}}{\varvec{o}}}\boldsymbol{ }{{\varvec{h}}}_{{\varvec{o}}{\varvec{u}}{\varvec{t}}}\left({{\varvec{T}}}_{{\varvec{c}}}-{{\varvec{T}}}_{{\varvec{a}}{\varvec{m}}}\right)+{{\varvec{A}}}_{{\varvec{c}}{\varvec{o}}}\boldsymbol{ }{\varvec{\sigma}}\boldsymbol{ }{{\varvec{\epsilon}}}_{{\varvec{c}}}\boldsymbol{ }\left({{\varvec{T}}}_{{\varvec{c}}}^{4}-{{\varvec{T}}}_{{\varvec{a}}{\varvec{m}}}^{4}\right).$$

${{\varvec{A}}}_{{\varvec{c}}{\varvec{o}}}$ denotes the outer cover area, ${{\varvec{h}}}_{{\varvec{o}}{\varvec{u}}{\varvec{t}}}$ is the outlet convection coefficient, ${\varvec{\sigma}}$ is the Stefan–Boltzmann constant, ${{\varvec{\epsilon}}}_{{\varvec{c}}}$ is the emissivity of the glass cover, ${T}_{c}$ is the cover temperature, and ${{\varvec{T}}}_{{\varvec{a}}{\varvec{m}}}$ is the ambient temperature.

Equation (6) shows the relationship of useful energy and receiver temperature^7^.

6 $${{\varvec{Q}}}_{{\varvec{u}}}={\varvec{h}}\boldsymbol{ }\boldsymbol{ }{{\varvec{A}}}_{{\varvec{r}}{\varvec{i}}}\boldsymbol{ }\left({{\varvec{T}}}_{{\varvec{r}}}-{{\varvec{T}}}_{{\varvec{f}}{\varvec{m}}}\right)$$

where ${T}_{r}$ is the receiver temperature, ${T}_{fm}$ is the mean fluid temperature, and *h* is the heat-transfer coefficient, where

7 $${\varvec{h}}=\frac{{\varvec{k}}\boldsymbol{ }\boldsymbol{ }{{\varvec{N}}}_{{\varvec{u}}}}{{{\varvec{D}}}_{{\varvec{r}}{\varvec{i}}}}$$

$k$ denotes the fluid thermal conductivity, and ${N}_{u}$ denotes the Nusselt number $(0.023 R{e}^{0.8} {Pr}^{0.33} \mathrm{for \; turbulent \; flow})$. $Re$ is the Reynolds number and $Pr$ is the Prandtl number, where $Pr=\mu \frac{{C}_{p}}{k}$.

In PTCs, two important performance indicators (PIs) are available. One indicator combines the ratio of useful energy to the available solar irradiation which is called thermal efficiency; it is defined by Eq. (8). The other indicator represents the exergetic performance of PTCs, and it is expressed by Eq. (9).

8 $${{\varvec{\eta}}}_{{\varvec{t}}{\varvec{h}}}=\frac{{{\varvec{Q}}}_{{\varvec{u}}}}{{{\varvec{Q}}}_{{\varvec{s}}}}$$

9 $${{\varvec{\eta}}}_{{\varvec{e}}{\varvec{x}}}=\frac{{{\varvec{E}}}_{{\varvec{u}}}}{{{\varvec{E}}}_{{\varvec{s}}}}$$

where ${E}_{{\varvec{u}}}$ and ${E}_{{\varvec{s}}}$ are defined as^7^.

10 $${{\varvec{E}}}_{{\varvec{u}}}={{\varvec{Q}}}_{{\varvec{u}}}-{\varvec{m}}\boldsymbol{ }{{\varvec{c}}}_{{\varvec{p}}}\boldsymbol{ }{{\varvec{T}}}_{{\varvec{a}}{\varvec{m}}}\boldsymbol{ }{\varvec{l}}{\varvec{n}}\left(\frac{{{\varvec{T}}}_{{\varvec{o}}{\varvec{u}}{\varvec{t}}}}{{{\varvec{T}}}_{{\varvec{i}}{\varvec{n}}}}\right)\; \mathrm{ and }\; {{\varvec{E}}}_{{\varvec{s}}}={{\varvec{Q}}}_{{\varvec{s}}}\left[1-\frac{4}{3}\boldsymbol{ }\left(\frac{{{\varvec{T}}}_{{\varvec{a}}{\varvec{m}}}}{{{\varvec{T}}}_{{\varvec{s}}{\varvec{u}}{\varvec{n}}}}\right)+\frac{1}{3}{\left(\frac{{{\varvec{T}}}_{{\varvec{a}}{\varvec{m}}}}{{{\varvec{T}}}_{{\varvec{s}}{\varvec{u}}{\varvec{n}}}}\right)}^{4}\right].$$

The areas ${A}_{ri}, {A}_{ro}, {A}_{ci}, and {A}_{co}$ are surface areas, and they were calculated based on the relation $A=\pi DL$. ${T}_{am}$ refers to the ambient temperature.

This nonlinear mathematical model is developed and implemented in MATLAB and then solved using an appropriate iterative technique. Finally, it is employed in the current MOO models.

The PTC efficiencies and energy storage depend on the thermal properties of the working fluids, such as thermal conductivity, pressure coefficient, density, and dynamic viscosity. Therefore, appropriate determination of the fluid properties is important in any PTC analysis or design process. Benoit et al.^33^ and other researchers in the field reviewed several thermal fluids and determined their correlations to obtain their thermal properties. Table 1 lists the nine thermal fluids that considered in the current optimization study as well as their physical and thermal properties. Column 1 indicates the fluid-type classification, i.e., either liquid or thermal. Column 2 lists all the fluids, Column 3 tabulates the equations for each fluid properties, and Column 4 provides the working temperature range corresponding to each fluid.

**Table 1:** Equations of the thermal fluid properties.

Type	Fluid	Property equation	T (K)
Liquidsa	Pressurized water^a^	${c}_{p}=0.01755{ T}^{2}-11.15 T+5931$ (11) $\kappa =-4.9\times {10}^{-6} {T}^{2}+0.00389 T-0.098$ (12) $\rho =-0.00225 {T}^{2}+0.895 T+928$ (13) $\mu =1.6\times {10}^{-8} {T}^{2}-1.52\times {10}^{-5} T+0.00371$ (14)	300–550
	Therminol VP-1^a^	${c}_{p}=1.058\times {10}^{-3} {T}^{3}-0.01458 {T}^{2}+9.192 T-156$ (15) $\kappa =-8.7\times {10}^{-24}{T}^{3}-1.786\times {10}^{-7} {T}^{2}+1.357\times {10}^{-5} T+0.147$ (16) $\rho =-2.25\times {10}^{-6} {T}^{3}+0.002511 {T}^{2}-1.746 T+1405$ (17) $\mu =7.542\times {10}^{-13} {T}^{4}-1.688\times {10}^{-9} {T}^{3}+1.403\times {10}^{-6} {T}^{2}-0.0005154 T+0.07097$ (18)	300–580
	Molten solar salt^33^	${c}_{p}=1443+0.172 (T-273.15)$ (19) $\kappa =0.443+1.9\times {10}^{-4}(T-273.15)$ (20) $\rho =2090-0.636 (T-273.15)$ (21) $\mu =2.2714\times {10}^{-2}-1.2\times {10}^{-4}\left(T-273.15\right)+2.281\times {10}^{-7}{\left(T-273.15\right)}^{2}-1.474\times {10}^{-10}{\left(T-273.15\right)}^{3}$ (22)	533–873
	Sodium^33^	${c}_{p}=1658.2-0.8479T+4.4541\times {10}^{-4} {T}^{2}-2.9926\times {10}^{6} {T}^{-2}$(23) $\kappa =124.67-0.11381 T+5.5226\times {10}^{-5} {T}^{2}-1.1842\times {10}^{-8} {T}^{3}$ (24) $\rho =219+275.32\left(1-\frac{T}{2503.7}\right)+511.58{\left(1-\frac{T}{2503.7}\right)}^{0.5}$ (25) $\mu =Exp\left(-6.4406-0.3958 ln\left(T\right)+\frac{556.835}{T}\right)$ (26)	371–1255
	Syltherm 800^15^	${c}_{p}=1.7075 T+1574.3$ (27) $\kappa =0.000188 T+0.138769$ (28) $\rho =-0.001 {T}^{2}-0.5325 T+919.01$ (29) $\mu =26.67122 {T}^{-1.917}$ (30)	425–630
Gases	Air^a^	${c}_{p}=1.573\times {10}^{-10} {T}^{4}-5.773\times {10}^{-7} {T}^{3}+0.0006741 {T}^{2}-0.09144 T+1002$ (31) $\kappa =9.518\times {10}^{-12} {T}^{3}-3.695\times {10}^{-8} {T}^{2}+8.724\times {10}^{-5} T+0.01312$ (32) $\rho =7.051\times {10}^{-13} {T}^{4}-2.875\times {10}^{-9} {T}^{3}+4.557\times {10}^{-6} {T}^{2}-0.003584 T+1.505$ (33) $\mu =-3.105\times {10}^{-22} {T}^{6}+1.49\times {10}^{-18} {T}^{5}-2.869\times {10}^{-15} {T}^{4}+2.833\times {10}^{-12} {T}^{3}-1.521\times {10}^{-9} {T}^{2}+4.527\times {10}^{-7} T-3.054\times {10}^{-5}$ (34)	300–1300
	Carbon dioxide^33^	${c}_{p}=651+0.918 T-3.32\times {10}^{-4} {T}^{2}$ (35) $\kappa =-1.1\times {10}^{-2}+9.74\times {10}^{-5} T-1.57\times {10}^{-8} {T}^{2}$ (36) $\rho =\frac{P}{{R}_{C{O}_{2}} T}$ (37) $\mu =5.94\times {10}^{-7}+5.3\times {10}^{-8} T-1.23\times {10}^{-11}{T}^{2}$ (38)	300–1300
	Helium^33^	${c}_{p}=5183+8.97\times {10}^{-3} T-2.58\times {10}^{-6} {T}^{2}$ (39) $\kappa =7.08\times {10}^{-2}+3.33\times {10}^{-4} T-3.91\times {10}^{-8} {T}^{2}$ (40) $\rho =\frac{P}{{R}_{He} T}$ (41) $\mu =8.64\times {10}^{-6}+4.23\times {10}^{-8} T-4.7\times {10}^{-12} {T}^{2}$ (42)	300–1300
	Hydrogen^33^	${c}_{p}=14994-1.72 T+1.72\times {10}^{-3} {T}^{2}$ (43) $\kappa =5.94\times {10}^{-2}+4.32\times {10}^{-4} T+3.6\times {10}^{-8} {T}^{2}$ (44) $\rho =\frac{P}{{R}_{H2} T}$ (45) $\mu =3.69\times {10}^{-6}+1.97\times {10}^{-8} T-3.08\times {10}^{-12} {T}^{2}$ (46)	300–1000

^a^Derived by the author.

## Model validation

**Figure 3:**
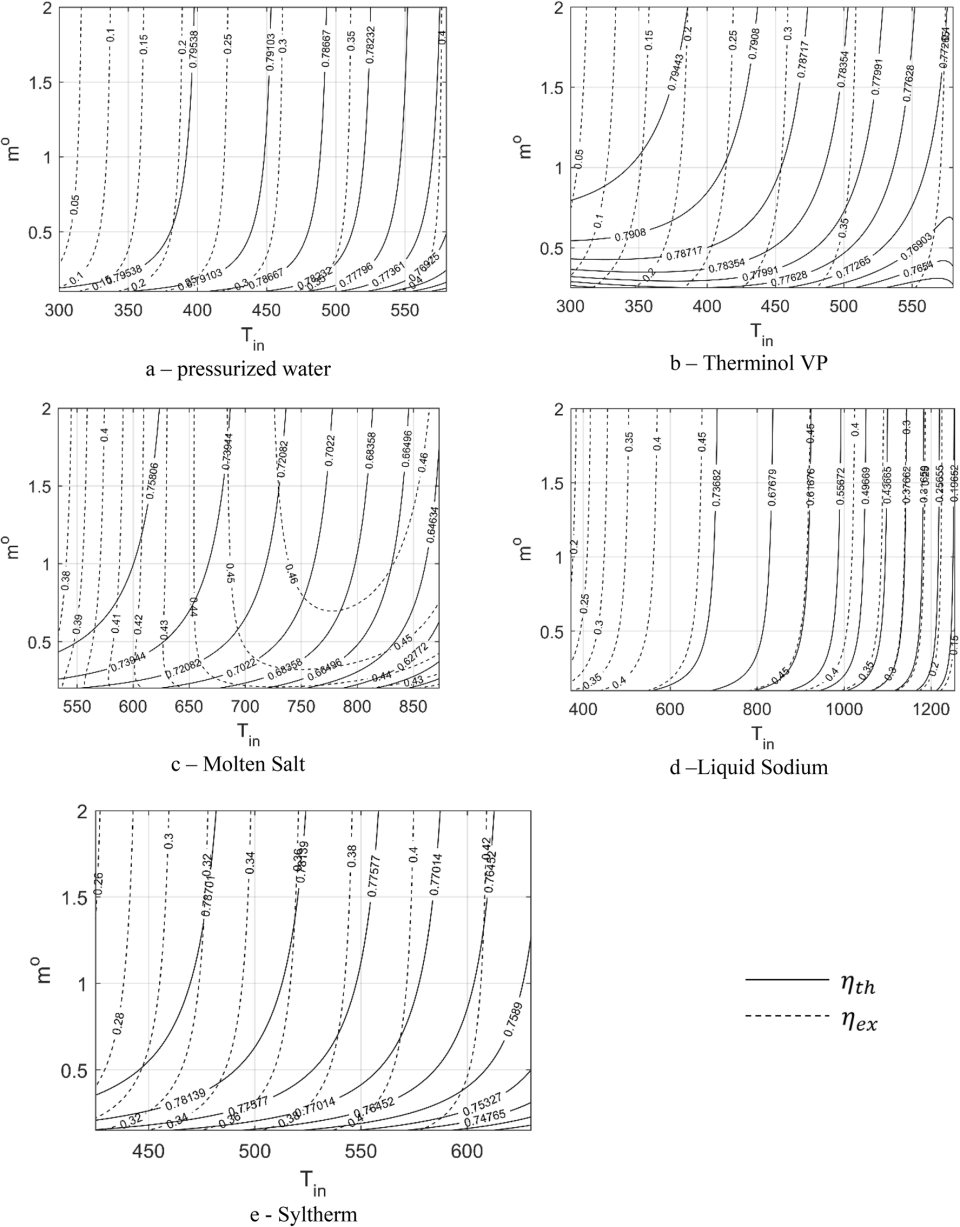
Level curves of the liquid performance in terms of the inlet temperature and mass flow rate.

To determine the accuracy of the present model, the results are compared to similar models from literature. The results are compared for air and molten salt at different inlet temperature, and they are listed in Table 3 (for air) and Table 4 (for molten salt). The PTC has the geometry and parameters given in Table 2. According to Tables 3 and 4, the present model demonstrates an accurate result (Fig. 3).

**Table 2:** Baseline design^7^.

Parameter	Value	Parameter	Value
Emissivity of receiver tube $\left({{\varvec{\epsilon}}}_{{\varvec{r}}}\right)$	0.095	Tube length $\left(L\right)$	12 m
Emissivity of glass cover $\left({{\varvec{\epsilon}}}_{{\varvec{c}}}\right)$	0.88	Reflector width $\left(W\right)$	5.8 m
Optical efficiency $\left({{\varvec{\eta}}}_{{\varvec{o}}{\varvec{p}}{\varvec{t}}}\right)$	0.9	Reflector radius $\left(f\right)$	1.71 m
Solar beam irradiation $\left({{\varvec{G}}}_{{\varvec{b}}}\right)$	800 $\frac{\mathrm{W}}{{\mathrm{m}}^{2}}$	Thermal conductivity $\left(k\right)$	0.628 $\frac{\mathrm{W}}{\mathrm{mK}}$
Sun temperature $\left({{\varvec{T}}}_{{\varvec{s}}{\varvec{u}}{\varvec{n}}}\right)$	5770 K	Density $\left(\rho \right)$	994
Ambient temperature $\left({{\varvec{T}}}_{{\varvec{a}}{\varvec{m}}}\right)$	300 K	Specific heat coefficient at constant pressure $\left({C}_{p}\right)$	4164 $\frac{\mathrm{J}}{\mathrm{kg} \mathrm{K}}$
Outlet convection coefficient $\left({{\varvec{h}}}_{{\varvec{o}}{\varvec{u}}{\varvec{t}}}\right)$	10 $\frac{\mathrm{W}}{{\mathrm{m}}^{2}\mathrm{K}}$	Dynamic viscosity $\left(\mu \right)$	$5.9 \times {10}^{-4} Pa.s$
Inlet temperature $\left({{\varvec{T}}}_{{\varvec{i}}{\varvec{n}}}\right)$	300 K

**Table 3:** Comparison with previous analyses from literature (for air).

${\mathrm{T}}_{\mathrm{in}} (\mathrm{K})$	Property	Value	Output parameter	Bellos 2017^7^	Present analytical model	Difference accuracy $(\left|\Delta \right|)$
300	$k$ $\left[\frac{\mathrm{W}}{\mathrm{mK}}\right]$	0.036	${\mathrm{T}}_{\mathrm{out}} [\mathrm{K}]$	511.2	513.8	2.6
	$\rho \left[\frac{\mathrm{kg}}{{\mathrm{m}}^{3}}\right]$	0.769	${\upeta }_{\mathrm{th}}$	0.7666	0.7842	0.0176
	${C}_{p}\left[\frac{\mathrm{J}}{\mathrm{kg K}}\right]$	1021	$\mathrm{h }[\frac{\mathrm{W}}{{\mathrm{m}}^{2}\mathrm{K}}]$	149	158.5	9.5
	$\mu \left[\mathrm{Pa s}\right]$	2.5e−5	${\upeta }_{ex}$	0.7881	0.7997	0.0116
400	$k$ $\left[\frac{\mathrm{W}}{\mathrm{mK}}\right]$	0.043	${\mathrm{T}}_{\mathrm{out}}[\mathrm{K}]$	603.3	605.77	2.47
	$\rho \left[\frac{\mathrm{kg}}{{\mathrm{m}}^{3}}\right]$	0.632	${\upeta }_{\mathrm{th}}$	0.7483	0.7687	0.0204
	${C}_{p}\left[\frac{\mathrm{J}}{\mathrm{kg K}}\right]$	1040	$\mathrm{h }[\frac{\mathrm{W}}{{\mathrm{m}}^{2}\mathrm{K}}]$	158	167.5	9.5
	$\mu \left[\mathrm{Pa s}\right]$	2.9e−5	${\upeta }_{ex}$	0.3229	0.3262	3.3e-3
500	$k$ $\left[\frac{\mathrm{W}}{\mathrm{mK}}\right]$	0.049	${\mathrm{T}}_{\mathrm{out}}[\mathrm{K}]$	692.3	695.3	3
	$\rho \left[\frac{\mathrm{kg}}{{\mathrm{m}}^{3}}\right]$	0.537	${\upeta }_{\mathrm{th}}$	0.7217	0.7451	0.0234
	${C}_{p}\left[\frac{\mathrm{J}}{\mathrm{kg K}}\right]$	1062	$\mathrm{h }[\frac{\mathrm{W}}{{\mathrm{m}}^{2}\mathrm{K}}]$	166	175.77	9.77
	$\mu \left[\mathrm{Pa s}\right]$	3.2e−5	${\upeta }_{ex}$	0.3771	0.3951	0.018
600	$k$ $\left[\frac{\mathrm{W}}{\mathrm{mK}}\right]$	0.054	${\mathrm{T}}_{\mathrm{out}}[\mathrm{K}]$	778.5	782.3	3.8
	$\rho \left[\frac{\mathrm{kg}}{{\mathrm{m}}^{3}}\right]$	0.467	${\upeta }_{\mathrm{th}}$	0.685	0.711	0.026
	${C}_{p}\left[\frac{\mathrm{J}}{\mathrm{kg K}}\right]$	1086	$\mathrm{h }[\frac{\mathrm{W}}{{\mathrm{m}}^{2}\mathrm{K}}]$	173	181.2	8.2
	$\mu \left[\mathrm{Pa s}\right]$	3.5e−5	${\upeta }_{ex}$	0.3995	0.4304	0.0309
700	$k$ $\left[\frac{\mathrm{W}}{\mathrm{mK}}\right]$	0.059	${\mathrm{T}}_{\mathrm{out}}[\mathrm{K}]$	862.4	866.8	4.4
	$\rho \left[\frac{\mathrm{kg}}{{\mathrm{m}}^{3}}\right]$	0.413	${\upeta }_{\mathrm{th}}$	0.6369	0.6638	0.0269
	${C}_{p}\left[\frac{\mathrm{J}}{\mathrm{kg K}}\right]$	1108	$\mathrm{h }[\frac{\mathrm{W}}{{\mathrm{m}}^{2}\mathrm{K}}]$	179	181.8	2.8
	$\mu \left[\mathrm{Pa s}\right]$	3.8e-5	${\upeta }_{ex}$	0.3972	0.4391	0.0419
800	$k$ $\left[\frac{\mathrm{W}}{\mathrm{mK}}\right]$	0.064	${\mathrm{T}}_{\mathrm{out}}[\mathrm{K}]$	943.9	949.1	5.2
	$\rho \left[\frac{\mathrm{kg}}{{\mathrm{m}}^{3}}\right]$	0.371	${\upeta }_{\mathrm{th}}$	0.576	0.605	0.029
	${C}_{p}\left[\frac{\mathrm{J}}{\mathrm{kg K}}\right]$	1129	$\mathrm{h }[\frac{\mathrm{W}}{{\mathrm{m}}^{2}\mathrm{K}}]$	184.8	188.5	3.7
	$\mu \left[\mathrm{Pa s}\right]$	4e−5	${\upeta }_{ex}$	0.3732	0.4262	0.053
900	$k$ $\left[\frac{\mathrm{W}}{\mathrm{mK}}\right]$	0.069	${\mathrm{T}}_{\mathrm{out}}[\mathrm{K}]$	1023	1028	5
	$\rho \left[\frac{\mathrm{kg}}{{\mathrm{m}}^{3}}\right]$	0.336	${\upeta }_{\mathrm{th}}$	0.5015	0.53	0.0285
	${C}_{p}\left[\frac{\mathrm{J}}{\mathrm{kg K}}\right]$	1148	$\mathrm{h }[\frac{\mathrm{W}}{{\mathrm{m}}^{2}\mathrm{K}}]$	189	192.8	3.8
	$\mu \left[\mathrm{Pa s}\right]$	4.3e−5	${\upeta }_{ex}$	0.3297	0.3921	0.062
1000	$k$ $\left[\frac{\mathrm{W}}{\mathrm{mK}}\right]$	0.073	${\mathrm{T}}_{\mathrm{out}}[\mathrm{K}]$	1100	1105	5
	$\rho \left[\frac{\mathrm{kg}}{{\mathrm{m}}^{3}}\right]$	0.307	${\upeta }_{\mathrm{th}}$	0.4126	0.4398	0.0272
	${C}_{p}\left[\frac{\mathrm{J}}{\mathrm{kg K}}\right]$	1164	$\mathrm{h }[\frac{\mathrm{W}}{{\mathrm{m}}^{2}\mathrm{K}}]$	194	196.9	2.9
	$\mu \left[\mathrm{Pa s}\right]$	4.5e−5	${\upeta }_{ex}$	0.2672	0.3377	0.07

**Table 4:** Comparison with previous analyses from literature (for molten salt).

${\mathrm{T}}_{\mathrm{in}} [\mathrm{K}]$	Property	Value	Output parameter	Bellos 2017 34	Present analytical model	Difference % $(\left|\Delta \right|\%)$
600	$k$ $\left[\frac{\mathrm{W}}{\mathrm{mK}}\right]$	0.506	${\mathrm{T}}_{\mathrm{out}}[\mathrm{K}]$	607	607.1	0.1
	$\rho \left[\frac{\mathrm{kg}}{{\mathrm{m}}^{3}}\right]$	1880	${\upeta }_{\mathrm{th}}$	0.7546	0.7665	0.0119
	${C}_{p}\left[\frac{\mathrm{J}}{\mathrm{kg K}}\right]$	1504	$\mathrm{h }[\frac{\mathrm{W}}{{\mathrm{m}}^{2}\mathrm{K}}]$	1492	1489.3	2.7
	$\mu \left[\mathrm{Pa s}\right]$	2.7e−3	${\upeta }_{ex}$	0.4288	0.4142	0.0146
700	$k$ $\left[\frac{\mathrm{W}}{\mathrm{mK}}\right]$	0.525	${\mathrm{T}}_{\mathrm{out}}[\mathrm{K}]$	706.6	706.8	0.2
	$\rho \left[\frac{\mathrm{kg}}{{\mathrm{m}}^{3}}\right]$	1816	${\upeta }_{\mathrm{th}}$	0.7263	0.7384	0.0121
	${C}_{p}\left[\frac{\mathrm{J}}{\mathrm{kg K}}\right]$	1521	$\mathrm{h }[\frac{\mathrm{W}}{{\mathrm{m}}^{2}\mathrm{K}}]$	1897	1885.5	11.5
	$\mu \left[\mathrm{Pa s}\right]$	1.6e−3	${\upeta }_{ex}$	0.4644	0.455	9.4e-3
800	$k$ $\left[\frac{\mathrm{W}}{\mathrm{mK}}\right]$	0.545	${\mathrm{T}}_{\mathrm{out}}[\mathrm{K}]$	806.2	806.3	0.1
	$\rho \left[\frac{\mathrm{kg}}{{\mathrm{m}}^{3}}\right]$	1752	${\upeta }_{\mathrm{th}}$	0.683	0.6955	0.0125
	${C}_{p}\left[\frac{\mathrm{J}}{\mathrm{kg K}}\right]$	1539	$\mathrm{h }[\frac{\mathrm{W}}{{\mathrm{m}}^{2}\mathrm{K}}]$	2142	2173.6	31.6
	$\mu \left[\mathrm{Pa s}\right]$	1.2e−3	${\upeta }_{ex}$	0.4734	0.4682	4.2e-3

## Results

### Parametric analysis

**Figure 4:**
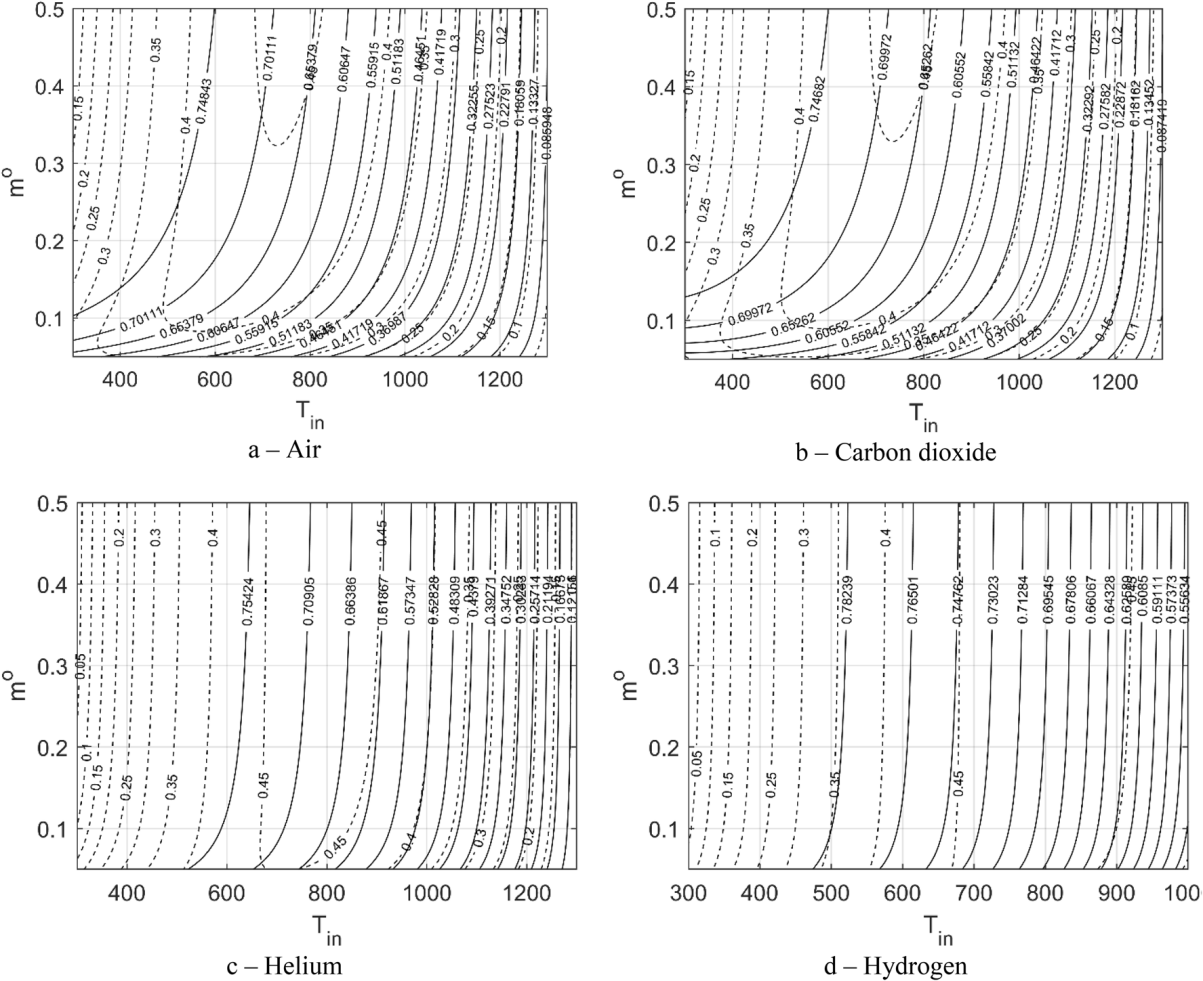
Level curves of the gase performance in terms of the inlet temperature and mass flow rate.

Before the MOO process is started, investigating the effect of inlet conditions $\left({T}_{in} and {m}^{o}\right)$ on the PTC PIs $\left({\eta }_{th} and {\eta }_{ex}\right)$ is necessary. This investigation will help determine which parameter is more effective for enhancing the PTC performance. Nine level curves are developed for the nine thermal fluids used in the present study. Error! Reference source not found. shows the level curves of the first five liquids, whereas Fig. 4 shows those of the four gases.

In general, inlet-mass flow rate is not as much significant as the inlet-temperature effect^7^. For liquids, the mass flow-rate effect is negligible at values of more than 2 $\frac{\mathrm{kg}}{{\mathrm{m}}^{3}\mathrm{s}}$; however, for gases its effect is negligible at values of more than 0.2 $\frac{\mathrm{kg}}{{\mathrm{m}}^{3}\mathrm{s}}$. Therefore, defining the inlet-mass flow rate as an optimization parameter (instead of defining it as a design variable) in the optimization process is better, which simplifies the optimization process and improves its efficiency. Irrespective of the minor effect of the mass flow rate, its effect is more significant in some fluids than that in others. In liquids, the mass flow rate is more effective in the case of Therminol, molten salt, and Syltherm than that in the case of water and liquid sodium. In gases, the effect of the mass flow rate is more remarkable in air and carbon dioxide than in helium and hydrogen. The increase in mass flow rate at low values can remarkably change the thermal-fluid performance compared with changing it to high values. In other words, as the mass flow rate increases, its effect becomes less important. Increasing ${m}^{o}$ simultaneously increases the thermal efficiency and decreases the exergetic efficiency.

On the other hand, the inlet-temperature effect is significant on both the thermal and exergetic efficiencies. A change in the inlet temperature does not result in similar changes in the efficiencies of all fluids. Table 5 lists the range of change of both the thermal and exergetic efficiencies according to each fluid. Air exhibits the maximum range of thermal-efficiency change, whereas water demonstrates the minimum range. Hydrogen archives a wider range of exergetic efficiency, and the minimum range is achieved by the molten salt. The increase in inlet temperature increases the exergetic efficiency and decreases the thermal efficiency for pressurized water, Therminol, sodium, Syltherm, and hydrogen; however, achieving this trend is different for molten salt, air, carbon dioxide and helium. The change in the inlet temperature in the liquids results in the changes in thermal and exergetic efficiencies up to $66\%$ and $42\%$, respectively (Table 5). In gases, the change in inlet temperature can result in more than $75\%$ change in the thermal efficiency and up to $46\%$ change in the exergetic efficiency (Table 5). This large range in the case of gases is due to their wide range of working temperature compared with liquids. The temperature ranges are defined according to the values listed in Table 1.

**Table 5:** Inlet-temperature effect on both thermal and exergetic efficiencies.

Type	Fluid	T [K]	Range of ${{\varvec{\eta}}}_{{\varvec{t}}{\varvec{h}}}$ %	${{\varvec{\eta}}}_{{\varvec{t}}{\varvec{h}}}$% Change	Range of ${{\varvec{\eta}}}_{{\varvec{e}}{\varvec{x}}}$ %	${{\varvec{\eta}}}_{{\varvec{e}}{\varvec{x}}}$% Change
Liquids	Pressurized water	300–550	76.17–79.99	3.82	0.3–40.27	39.97
	Therminol VP-1	300–580	66.69–79.92	13.23	0.8–43.47	42.67
	Molten solar salt	533–873	50.02–77.96	27.94	36.88–46.85	9.97
	Liquid Sodium	371–1255	13.65–79.71	66.06	11.24–47.06	35.82
	Syltherm 800	425–630	71.32–79.33	8.01	25.34–43.92	18.58
Gases	Air	300–1300	3.86–79.58	75.72	3.21–45.64	42.43
	Carbon dioxide	300–1300	4.03–79.39	75.36	3.35–45.62	42.27
	Helium	300–1300	7.64–79.94	72.3	2.37–46.78	44.41
	Hydrogen	300–1000	52.16–79.98	27.82	0.86–46.95	46.09

We compare the performance among the fluids at a certain temperature so that one temperature can be selected e.g., 500 K, for application to all liquids and gases. According to the list in Table 6, water has the best thermal efficiency, followed by liquid sodium. Meanwhile, air has the highest exergetic efficiency followed by carbon dioxide. The last column in Table 6 denotes the average sum of both thermal and exergetic efficiencies as PI. Air is found to demonstrate the best performance in terms of average of the sum of the thermal and exergetic efficiencies followed by helium. Molten Salt achieves the minimum average efficiency.

**Table 6:** Comparison of the performance among the fluids at selected inlet temperature (500 K).

Type	Fluid	${\eta }_{th}$%	${\eta }_{ex}$%	$\frac{\left({\eta }_{th}+{\eta }_{ex}\right)}{2}\%$
Liquids ${m}^{o}=2\frac{\mathrm{kg}}{\mathrm{s}}$	Pressurized water	78.58	34.01	56.295
	Therminol VP-1	78.39	34.21	56.3
	Molten solar salt	78.16	34.32	56.24
	Liquid Sodium	78.54	34.57	56.555
	Syltherm 800	78.48	34.18	56.33
Gases ${m}^{o}=0.2\frac{\mathrm{kg}}{\mathrm{s}}$	Air	74.42	39.45	56.935
	Carbon dioxide	73.94	39.36	56.65
	Helium	78.07	35.55	56.81
	Hydrogen	78.44	34.46	56.45

### MOO of PTC

MOO is based on the maximization or minimization of more than one objective function. When two objectives are used, the best set of points is obtained using what is called as pareto optimal or pareto front (Fig. 1). The Pareto-optimal technique was first proposed by Vifredo Pareto^34^ in 1906. Since then, it has been used to solve several optimization problems. In general, it represents the locus of points ${x}^{*}$ such that no feasible point *x* exists with $F\left(x\right)\le F({x}^{*})$^35^, where *F* is the objective function to be minimized. Madeira et al.^36^ used this technique for MOO of a sandwiched composite plate. They used the direct multisearch technique to minimize the weight of the plate and maximize the loss factor. The results were compared with those obtained using GA. The Pareto front was developed for simply supported sandwich beams and plates. Similar research was conducted by the same authors using the Pareto-front technique for MOO of laminated composite panels^37^. According to Madeira et al., Pareto optimal is efficient in obtaining an optimal solution. However, MOO is more powerful than other optimization techniques, some challenges and limitations are associate with using MOO. MOO is more difficult than the conventional optimization methods. If more than three objective functions are included, pareto front cannot be obtained.

Selecting a proper optimization technique for an optimization problem and defining the proper optimization model are important. Several optimization techniques can be found in the literature. GA is classified as a global nongradient optimization algorithm based on natural-selection process^38^. It was first proposed by Holland^39^ in 1975. Since then, it has been improved in several means to increase its speed and efficiency. Guo and Yang^40^ introduced a modified version of simple or conventional GA. In the present study, GA is employed for MOO.

The optimization problem is defined as the process of maximizing both the thermal and exergetic efficiencies by changing the inlet temperature, PTC length, and absorber-tube inner and outer diameters subject to a constant volume, as expressed in Eq. (47). Selection of a constant volume is important because the main objective of this study is to enhance the PCT performance without any extra cost or material. Therefore, constant volume in the present optimization model refers to obtaining additional performance without incurring additional cost.

47 $$ \begin{gathered} {\text{Find}}\,{\text{the}}\,{\text{design}}\,{\text{variable}}\,{\text{vector}}\,\vec{x} = \left( {\overline{{T_{in} }} ,\overline{L}, \overline{{D_{ri} }} ,\overline{{D_{ro} }} ,\overline{{D_{co} }} , \overline{{D_{ci} }} } \right)^{T} ,{\text{which}} \hfill \\ {\mathbf{Maximizes}} \quad \left( {\eta_{th} \left( {\vec{x}} \right), \eta_{ex} \left( {\vec{x}} \right)} \right) \hfill \\ {\mathbf{Subject}} {\mathbf{to}}\quad \overline{V} - 1 = 0 \hfill \\ x_{i}^{L} \le x_{i} \le x_{i}^{U} , \quad i = 1,2, \ldots ,N \hfill \\ x^{L} = \left\{ {\overline{{T_{in}^{min} }} , 0.8, 0.8, 0.8, 0.8, 0.8} \right\} \hfill \\ x^{U} = \left\{ {\overline{{T_{in}^{max} }} , 1.2, 1.2, 1.2, 1.2, 1.2} \right\}. \hfill \\ \end{gathered} $$

${D}_{ri}$, ${D}_{ro}$, ${D}_{co}$, and ${D}_{ci}$ are the receiver inner diameter, receiver outer diameter, cover outer diameter, and cover inner diameter, respectively. The parabolic trough length and diameters are changed from 0.8 to 1.2 with respect to the baseline design during the optimization. The bar over these variables indicates that their values are normalized with respect to the benchmark values listed in Table 2. The side constraints of inlet temperature ${T}_{in}$ follow the boundaries listed in Table 1. To improve the optimization performance and measure the optimization-process efficiency, the objective function and both the design variables and constraints are normalized with respect to a baseline design that is selected from the literature^7^.

**Figure 5:**
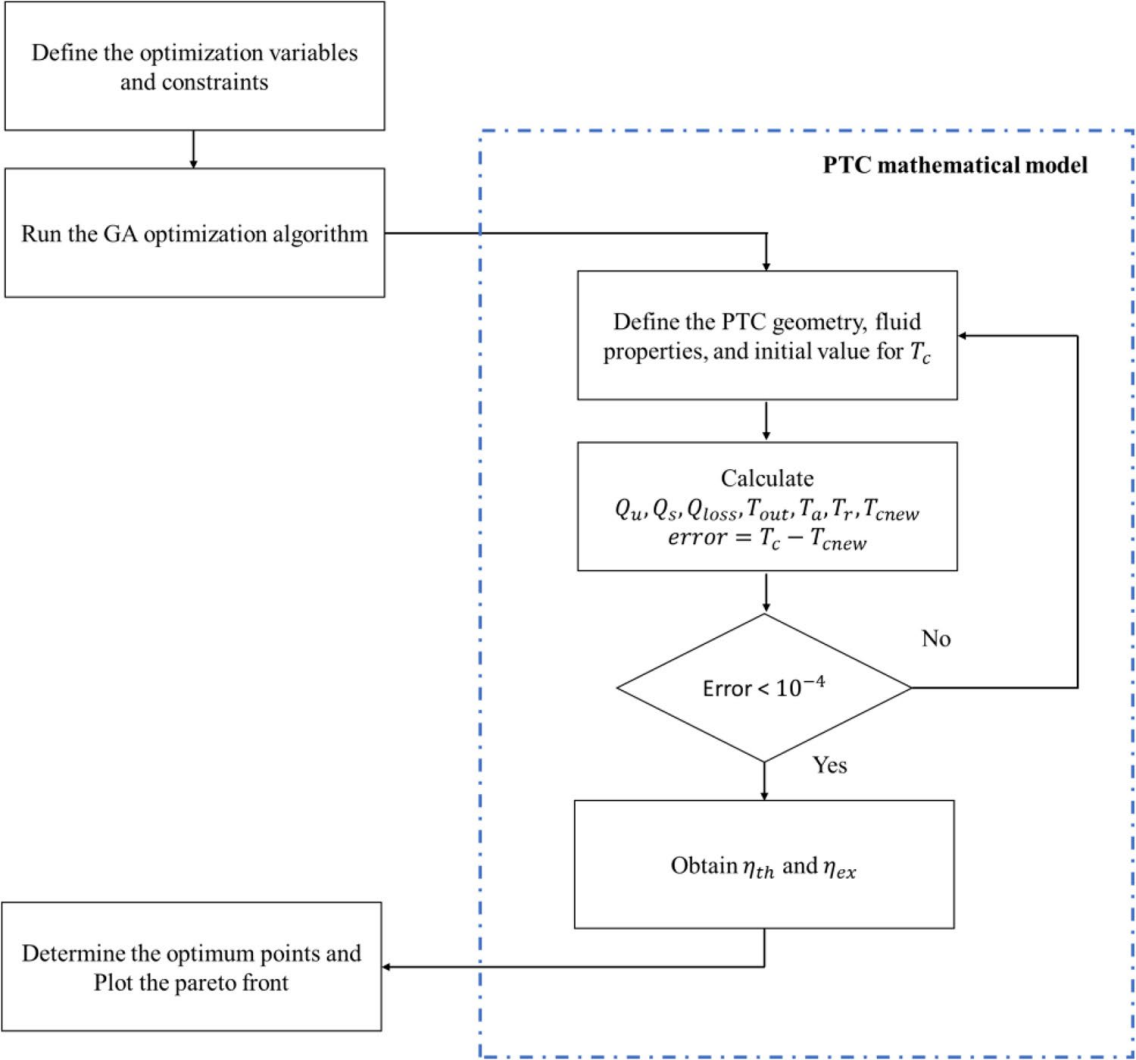
Flowchart for the optimization process.

*V* refers to the PTC material volume and it is normalized with respect to the baseline volume (Appendix A). The volume of the baseline design is calculated as $3.662 {\mathrm{m}}^{3}$, and it is kept constant throughout the optimization process. Figure 5 shows a flowchart for the design process.

**Figure 6:**
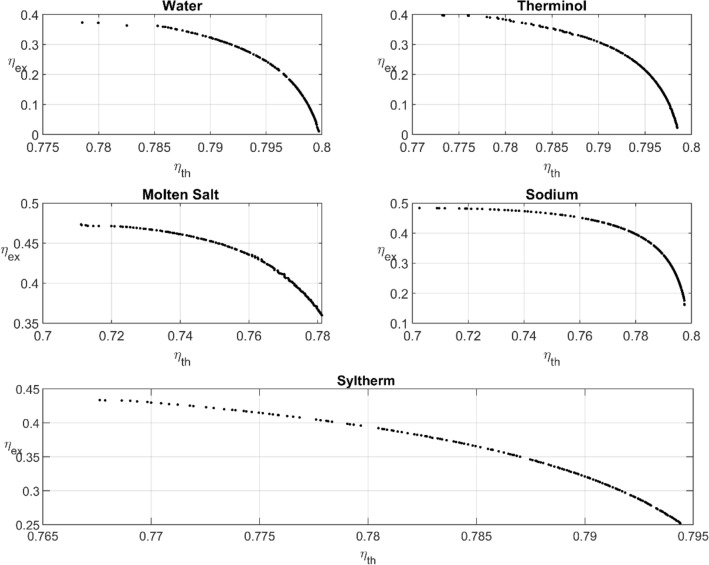
Pareto fronts of the liquid thermal fluids.

**Figure 7:**
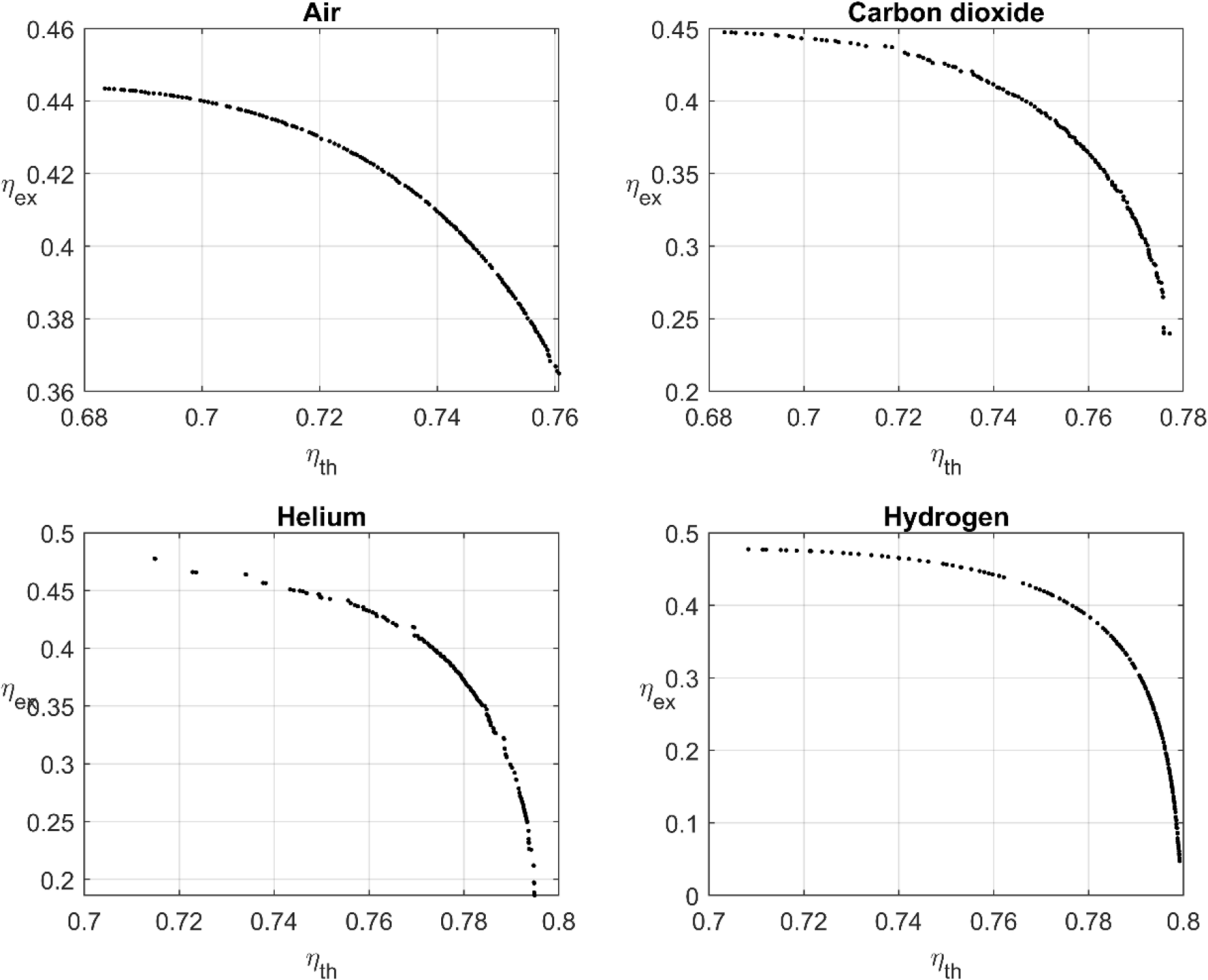
Pareto fronts of the gas thermal fluids.

Notice that both the thermal and exergetic efficiencies are defined as objective functions, in the present MOO. When two objective functions are used the optimization algorithm usually define them in the form ${\varvec{f}}\left(\overrightarrow{{\varvec{x}}}\right)=\boldsymbol{\alpha } {{\varvec{\eta}}}_{{\varvec{t}}{\varvec{h}}}\left(\overrightarrow{{\varvec{x}}}\right)- \left(1-\boldsymbol{\alpha }\right) {{\varvec{\eta}}}_{{\varvec{e}}{\varvec{x}}}\left(\overrightarrow{{\varvec{x}}}\right)$^41^, where $\boldsymbol{\alpha }$ is a weight function that determines the weight of each objective in the optimization process. The weight of the objective function is changed throughout the optimization based on the preferences of the optimization method and to obtain the pareto plots (Figs. 6, 7).

Nine optimization problems are solved for the nine thermal fluids. The results are shown in Figs. 6 and 7 for the liquids and gases, respectively. According to Table 7, the pressurized-water thermal efficiency can reach a maximum value of as high as $79.97\%$ with a corresponding exgergetic efficiency of $1.17\%$. The exergetic efficiency can also reach a maximum value of as high as $37.29 \%$ with a corresponding thermal efficiency of $77.85\%$. This performance is generally better than the baseline design performance. The maximum thermal efficiency $\left(79.97\%\right)$ is obtained by water; however, the maximum exergetic efficiency $\left(48.37\%\right)$ is achieved by the sodium liquid. The best performance for both thermal and exergetic efficiencies is achieved by the molten salt. For the molten salt the maximum thermal efficiency is $79.75\%$ with a corresponding exergetic efficiency of $35.99\%$, whereas the maximum exergetic efficiency is $47.34\%$ with a corresponding thermal efficiency $71.11\%$.

**Table 7:** PTC optimum performance.

Type	Fluid	Design point	$\left({\eta }_{th},{\eta }_{ex}\right)\%$	$\frac{\left({\eta }_{th}+{\eta }_{ex}\right)}{2}\%$
Liquids ${m}^{o}=2\frac{\mathrm{kg}}{\mathrm{s}}$	Pressurized water	$\left(1.004, \mathrm{1.135,0.863,1.013,1.083,1.191}\right)$	$\left(79.97, 1.17\right)$	40.57
		$\left(1.607, \mathrm{1.002,0.907,0.833,1.106,1.093}\right)$	$\left(78.99, 32.32\right)$	55.655
		$\left(1.797, \mathrm{0.995,0.936,1.084,1.015,1.054}\right)$	$\left(77.85, 37.29\right)$	57.57
	Therminol VP-1	$\left(1.002, \mathrm{1.041,0.845,0.895,0.886,0.916}\right)$	$\left(79.85, 2.23\right)$	41.04
		$\left(1.7, \mathrm{1.014,0.823,0.863,0.854,0.862}\right)$	$\left(78.52, 35.26\right)$	56.89
		$\left(1.899, \mathrm{0.938,1.067,1.008,1.009,0.94}\right)$	$\left(77.32, 39.68\right)$	58.5
	Molten solar salt	$\left(1.727, \mathrm{0.977,0.923,0.904,1.019,0.995}\right)$	$\left(78.12, 35.99\right)$	57.055
		$\left(2.245, \mathrm{0.951,1.094,0.869,1.165,1.073}\right)$	$\left(75.04, 45.06\right)$	60.05
		$\left(2.608, \mathrm{0.93,1.077,0.877,1.14,1.035}\right)$	$\left(71.11, 47.34\right)$	59.225
	Liquid Sodium	$\left(1.206, \mathrm{0.966,1.049,0.939,0.987,0.919}\right)$	$\left(79.75, 16.11\right)$	47.93
		$\left(2.333, \mathrm{1.099,1.164,0.824,0.959,0.883}\right)$	$\left(74.98, 46.47\right)$	60.725
		$\left(2.755, \mathrm{1.099,1.164,0.819,0.9588,0.8826}\right)$	$\left(70.25, 48.37\right)$	59.31
	Syltherm 800	$\left(1.402, \mathrm{1.087,0.826,0.808,0.903,0.942}\right)$	$\left(79.44, 25.22\right)$	52.33
		$\left(1.863, \mathrm{1.087,0.8258,0.803,0.905,0.943}\right)$	$\left(78.05, 39.21\right)$	58.63
		$\left(2.093, \mathrm{1.099,0.8309,0.825,0.903,0.953}\right)$	$\left(76.76, 43.35\right)$	60.055
Gases ${m}^{o}=0.2\frac{\mathrm{kg}}{\mathrm{s}}$	Air	$\left(1.501, \mathrm{0.967,0.924,0.97,1.0114,0.999}\right)$	$\left(76.06, 36.49\right)$	56.275
		$\left(1.962, \mathrm{0.968,0.919,0.97,1.0177,1.008}\right)$	$\left(72.02, 42.96\right)$	57.49
		$\left(2.256, \mathrm{0.969,0.916,0.967,1.021,1.012}\right)$	$\left(68.34, 44.35\right)$	56.345
	Carbon dioxide	$\left(1.0615, \mathrm{0.945,0.956,0.9196,1.062,1.014}\right)$	$\left(77.71, 23.98\right)$	50.845
		$\left(1.844, \mathrm{0.954,0.9055,0.885,1.084,1.049}\right)$	$\left(73.54, 42.04\right)$	57.79
		$\left(2.335, \mathrm{0.8296,1.089,0.86,1.182,0.966}\right)$	$\left(68.33, 44.74\right)$	56.535
	Helium	$\left(1.203, \mathrm{1.0797,1.055,1.0699,1.0599,1.118}\right)$	$\left(79.49, 18.62\right)$	49.055
		$\left(2.117, \mathrm{1.105,0.998,0.882,1.12,1.154}\right)$	$\left(75.56, 44.14\right)$	59.85
		$\left(2.582, \mathrm{1.017,1.046,0.803,0.953,0.865}\right)$	$\left(71.48, 47.78\right)$	59.63
	Hydrogen	$\left(1.034, \mathrm{0.977,0.997,0.963,1.025,0.999}\right)$	$\left(79.92, 4.97\right)$	42.445
		$\left(2.285, \mathrm{0.887,0.952,0.87,1.051,0.929}\right)$	$\left(75, 45.64\right)$	60.32
		$\left(2.663, \mathrm{0.889,0.951,0.868,1.0517,0.931}\right)$	$\left(70.83, 47.74\right)$	59.285

In general, the optimum performance of gases is lower than that of liquids; however, the key characteristic of gases is that they can be used in a wide range of working temperature, which indicates their advantage. The maximum thermal efficiency $\left(79.97\%\right)$ is obtained by water, whereas the maximum exergetic efficiency $\left(47.78\%\right)$ is achieved by helium. Helium demonstrates the best performance in terms of both thermal and exergetic efficiencies. Table 7 lists the best three points obtained from MOO in all nine fluids. It tabulates the maximum point according to the thermal and exergetic temperature perspective and the average optimization point. The last column, in Table 7, indicates the average of the sum of both the thermal and exergetic efficiencies as PI. The PI values in the last column provides a further prospective for the fluid performance. Thus, according to Table 7, the maximum value of PI is obtained for liquid sodium followed by hydrogen and Syltherm.

## Conclusions and future work

The purpose of the present study is to investigate and enhance the performance of PTCs. A mathematical model of PTC performance is developed and implemented using a MATLAB code. By applying MOO, an optimization model is developed for the design of PTCs. Nine fluids are considered, which include five liquids and four gases. The fluids are pressurized water, Therminol VP1, molten salt, liquid sodium, Syltherm, air, carbon dioxide, helium and hydrogen. All optimizations are conducted using MATLAB, and Pareto front curves are obtained.

Before the optimization process is started, a parametric study is conducted, and the level curves for each fluid are obtained. In the parametric analysis, the author studies the effect of changing both the inlet-mass flow rate and inlet temperature on each fluid performance. The inlet temperature is found to be more effective than the inlet-mass flow rate in improving PTCs performance in terms of thermal and exergetic efficiencies. Thus, in the optimization, the inlet temperature is used as an optimization variable while the inlet-mass flow rate is considered as an optimization parameter.

In MOO, the objective (cost) function is defined as the thermal and exergetic efficiencies. The design variables are the inlet temperature and receiver inlet and outlet diameters. The PTC material volume is kept constant during the optimization to enhance the PTC performance without introducing any additional cost. The developed multioptimization model is efficient in improving the PTC performance. Pareto fronts are obtained for all the used fluids.

So, the objectives of the present study can be summarized as following,

- Conduct a *parametric analysis* using *level curves to investigate the effect of PTC parameters and variable on its performance*.
- Develop an original *multiobjective optimization* model for the design of *PTCs* by which the objective functions are the *thermal and exergetic efficiencies* and the PTC *material volume* is kept constant throughout the optimization process
- Investigate nine *working fluids*, and the Pareto front (locus of optimal points) is obtained for each fluid.

The present parametric study and optimization model can be used directly by researchers in the field to study and improve PTCs performance. A researcher can obtain her/his preferred optimal point for the analysis and design of PTCs using the present Pareto fronts.

## Supplementary Information

Supplementary Information.

## Data Availability

The datasets generated and/or analyzed during the current study are not publicly available, because it includes MATLAB codes that have been developed by the author, but are available from the corresponding author on reasonable request.

## List of symbols

${A}_{a}$: Trough aperture area $({\mathrm{m}}^{2})$
${A}_{r}$: Area of the receiver $({\mathrm{m}}^{2})$
${C}_{p}$: Specific heat capacity $(\frac{\mathrm{J}}{\mathrm{kg} \mathrm{K}})$
${D}_{co}$: Outer cover diameter (m)
${D}_{ci}$: Inner cover diameter (m)
${D}_{ri}$: Inner receiver diameter (m)
${D}_{ro}$: Outer receiver diameter (m)
${E}_{u}$: Useful exergetic gain (W)
${E}_{s}$: Solar exergy input (W)
L: Parabolic trough length (m)
${m}^{o}$: Mass flow rate $(\frac{\mathrm{kg}}{\mathrm{s}})$
$${T}_{a}$$: Ambient temperature (K)
$${T}_{in}$$: Inlet temperature (K)
$${T}_{out}$$: Outlet temperature (K)
$${T}_{r}$$: Receiver temperature (K)
${T}_{fm}$: Mean fluid temperature
$${T}_{sun}$$: Sun temperature (K)
$${Q}_{u}$$: Useful energy (W)
$${Q}_{loss}$$: Heat loss (W)
${Q}_{s}$: Available solar energy
$\sigma$: Stefan-Boltzmann constant $\left(\frac{\mathrm{W}}{{\mathrm{m}}^{2}{\mathrm{K}}^{4}}\right)$
${\epsilon }_{c}$: Emissivity of glass cover
${\epsilon }_{r}$: Emissivity of receiver tube
$${\eta }_{th}$$: Thermal efficiency
$${\eta }_{ex}$$: Exergetic efficiency
$${\eta }_{o}$$: Optical efficiency
