# Supplementary material for: Multiobjective design optimization of parabolic trough collectors
Source: Sci Rep. 2022 Nov 19;12:19964. doi: 10.1038/s41598-022-24305-3 (PMC9675770; doi:10.1038/s41598-022-24305-3)
Supplement: Supplementary file 1 — Supplementary Information. [file 41598_2022_24305_MOESM1_ESM.pdf]

Multiobjective Design Optimization of Parabolic Trough Collectors

Mohamed Mahran Kasem

Aerospace Engineering Department, Cairo University, Giza 12613, Egypt,
School of Engineering and Applied Science, Nile University, Shaikh Zayed City 12588, Egypt
Email: mohamed.kasem@cu.edu.eg; mkasem@nu.edu.eg

Appendix A – Material volume of PTC

PTC material volume (V_{ptc}) = cover volume + receiver volume + reflector volume

cover tube volume

$$V_c = A_{ci}(D_{co} - D_{ci})$$

receiver tube volume

$$V_r = A_{ri}(D_{ro} - D_{ri})$$

Reflector thickness

$$t_{ref} = 0.004$$

Reflector perimeter [40]

$$P_{ref} = \frac{1}{2}\sqrt{W^2 + 16f^2} + \frac{W^2}{8f}\log\left(\frac{4f + \sqrt{W^2 + 16f^2}}{W}\right)$$

Reflector volume

$$V_{ref} = P_{ref}\, L\, t_{ref}$$

$$V_{ptc} = V_c + V_r + V_{ref}$$
